# Quantum double-double-slit experiment with momentum entangled photons

**DOI:** 10.1038/s41598-020-68181-1

**Published:** 2020-07-10

**Authors:** Manpreet Kaur, Mandip Singh

**Affiliations:** Department of Physical Sciences, Indian Institute of Science Education and Research (IISER) Mohali, Sector-81, Mohali, 140306 India

**Keywords:** Optics and photonics, Physics

## Abstract

Double-double-slit thought experiment provides profound insight on interference of quantum entangled particles. This paper presents a detailed experimental realisation of quantum double-double-slit thought experiment with momentum entangled photons and theoretical analysis of the experiment. Experiment is configured in such a way that photons are path entangled and each photon can reveal the which-slit path information of the other photon. As a consequence, single photon interference is suppressed. However, two-photon interference pattern appears if locations of detection of photons are correlated without revealing the which-slit path information. It is also shown experimentally and theoretically that two-photon quantum interference disappears when the which-slit path of a photon in the double-double-slit is detected.

## Introduction

Wave nature of light was first experimentally demonstrated by the famous Young’s double-slit experiment^1,2^. In quantum physics, light is quantised in the form of energy quanta known as photon. According to the statement of P.A.M. Dirac, “Each photon interferes only with itself”^3^. This self interference of a photon is a consequence of quantum superposition principle. If photons are incident on a double-slit one by one then the interference pattern of a photon gradually emerges. Where detection of each photon corresponds to a point on the screen. Young’s double-slit experiment provides profound insight on the wave-particle duality if it is imagined for individual particles^4^. Interference pattern of a single particle is not formed if the path information of a particle i.e. a slit through which a particle has passed, is known. According to Copenhagen interpretation, an observation on the quantum superposition of paths of a particle corresponds to a measurement that collapses quantum superposition therefore, no interference pattern is formed. On the other hand, what happens if we modify the experiment in such a way that the which-path information of a particle is not available during its passage through a double-slit but can be obtained even after its detection. In this case, the which-path information can be carried out by the quantum state of another particle if total quantum state of particles is an entangled quantum state. By knowing its path by a measurement, the path information of the other particle is immediately determined. Because of path revealing quantum entanglement of particles the single particle interference is suppressed. However, quantum interference can be recovered even after completion of experiment by making correlated selection of measurement outcomes.

The first experiment to show the interference of light with very low intensity in the Young’s double-slit experiment was performed in 1909 by G.I. Taylor^5^. Interesting experiments showing the Young’s double-slit interference are performed with neutrons from the foundational perspective of quantum mechanics^6^, with electron beams^7^ and with a single electron passing through a double-slit^8–10^. Recently, a first experimental demonstration of interference of antiparticles with a double-slit is reported^11^. Interference of macromolecules is the subject of great interest in the quest to realise quantum superposition of mesoscopic and macroscopic objects^12,13^. In this context, number of interesting experiments have been performed to produce a path superposition of large molecules similar to the double-slit type interference experiments^14–16^.

The main concept of a quantum single double-slit experiment was extended to a quantum double-double-slit thought experiment by Greenberger, Horne and Zeilinger^17^ to provide foundational insight on the multiparticle quantum interference. In their paper. they have considered two double-slits and a source of particles placed in the middle of double-slits. Each particle is detected individually after it traverses a double-slit. Quantum entanglement of particles appears naturally in their considerations^18,19^ and it is shown, when single particle interference disappears and two-particle interference appears. An experimental realisation of quantum double-double-slit thought experiment showing a two-photon interference has been demonstrated with quantum correlated photons produced by spontaneous parametric down conversion (SPDC) process^20^. However, in this paper, we present a detailed experimental realisation of the quantum double-double-slit thought experiment with momentum entangled photon pairs, where a virtual double-double-slit configuration is realised with two Fresnel biprisms. This paper provides a detailed conceptual, theoretical and experimental analysis of the quantum double-double-slit experiment. In addition, an experiment of detection of a which-slit path of a photon is presented where it is shown that the two-photon interference disappears when a which-slit path of a photon is detected.

In this paper, experiments are presented in the context of a quantum double-double-slit thought experiment. However, experiments of foundational significance with polarization entangled photons^21–23^ and momentum entangled photons^24^ have been intensively studied. In addition, interesting experiments on delayed choice path erasure^25–30^ and two-photon interference^31–40^ are performed. Similar experiments have been proposed with Einstein–Podolsky–Rosen (EPR) entangled pair of atoms^41^.

## Quantum double-double-slit experiment

**Figure 1:**
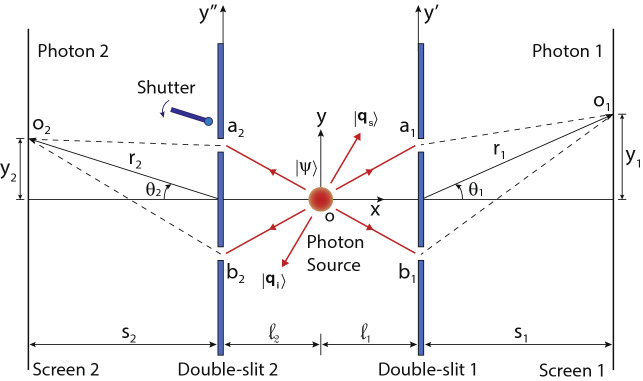
A schematic diagram of a double-double-slit experiment. Photons are individually detected on screens after they pass through the double-slits separately. Which-slit path information of photons can be detected by blocking any single slit by closing the shutter.

Quantum double-double-slit experiment consists of two double-slits and a source of photon pairs. In this experimental situation, a single photon passes through each double-slit and detected individually on screens positioned behind the double-slits as shown in Fig. 1. However, interference of photons depends on the quantum state of two photons. To understand quantum interference of two photons in a double-double-slit experiment, consider a source is producing photons in pairs and both the photons have same linear polarisation. Double-slit 1 and double-slit 2 are aligned parallel to *y*-axis and positioned at distances $l_{1}$ and $l_{2}$, respectively along the *x*-axis from the source. Single slits $a_{1}$ and $b_{1}$ of double-slit 1 are separated by a distance $d_{1}$ and single slits $a_{2}$ and $b_{2}$ of double-slit 2 are separated by a distance $d_{2}$ as shown in Fig. 1 where each slit width is considered to be infinitesimally small. A single photon of a photon pair is detected on screen 1, which is positioned at a distance $s_{1}$ from double-slit 1 and a second photon is detected on screen 2, which is positioned at a distance $s_{2}$ from double-slit 2. There are four different possible paths by which photons can arrive at the respective screens i.e. a photon can arrive at a point $o_{1}$ on screen 1 via double-slit 1 and the other photon can arrive at a point $o_{2}$ on screen 2 via double-slit 2. Therefore, possible paths of photons are (i) a first photon can pass through slit $a_{1}$ and the second photon can pass through slit $a_{2}$, or (ii) a first photon can pass through slit $b_{1}$ and the second photon can pass through slit $b_{2}$, or (iii) a first photon can pass through slit $a_{1}$ and the second photon can pass through slit $b_{2}$, or (iv) a first photon can pass through slit $b_{1}$ and the second photon can pass through slit $a_{2}$. Since all the possible paths are indistinguishable and not revealing any which-path information therefore, total amplitude $A_{12}$ to find a photon at $o_{1}$ and a photon at $o_{2}$ together is a quantum superposition of all the possible paths, which can be successively written as

1 $$\begin{aligned} A_{12}= & {} \langle o_{2}|a_{2}\rangle t_{a2} \langle a_{2}| \langle o_{1}|a_{1}\rangle t_{a1}\langle a_{1}|\psi \rangle + \langle o_{2}|b_{2}\rangle t_{b2} \langle b_{2}| \langle o_{1}|b_{1}\rangle t_{b1}\langle b_{1}|\psi \rangle \nonumber \\&+\langle o_{2}|b_{2}\rangle t_{b2} \langle b_{2}| \langle o_{1}|a_{1}\rangle t_{a1}\langle a_{1}|\psi \rangle +\langle o_{2}|a_{2}\rangle t_{a2} \langle a_{2}| \langle o_{1}|b_{1}\rangle t_{b1}\langle b_{1}|\psi \rangle \end{aligned}$$

where $t_{a1}$, $t_{b1}$, $t_{a2}$, $t_{b2}$ are amplitudes of transmission of slits $a_{1}$, $b_{1}$, $a_{2}$, $b_{2}$, respectively. Quantum states $|a_{1}\rangle$, $|b_{1}\rangle$ $|a_{2}\rangle$, $|b_{2}\rangle$ are position space basis states of locations on the slits on double-slit 1 and double-slit 2, respectively where a photon can be found. Similarly, $|o_{1}\rangle$ and $|o_{2}\rangle$ are the position space basis states of locations on the screens. However, position basis states corresponding to points on each double-slit and a screen form a different basis set such that $\langle o_{1}|a_{1}\rangle$ represents the amplitude of transmitted photon to go from slit $a_{1}$ to a location $o_{1}$ on screen 1. Same terminology is applied for other amplitudes in Eq. 1.

Further, consider photon pairs produced by a source of finite size are emitted in opposite directions w.r.t. each other such that they are momentum entangled, their net momentum is zero and momentum of each photon is definitely unknown. Consider the spatial extension of source is much smaller than the slit separation but large to produce momentum entanglement. As a consequence of momentum entanglement, if a photon passes through slit $a_{1}$ then the other photon passes through slit $b_{2}$ and if a photon passes through slit $b_{1}$ then the other photon passes through slit $a_{2}$ after their transmission through the slits. For momentum entangled photons, both these possibilities are quantum superimposed, as result of it both the photons are path entangled via the slits and first two terms in the summation of Eq. 1 become zero. The last two terms in the summation are due to path entanglement via the slits, these two amplitudes interfere with each other and produce a two-photon interference of momentum entangled photons. When all four slits are opened, a two-photon path information is not revealed and a two-photon interference can be observed by recording detection locations of a photon corresponding to a particular location of detection of other photon on the other screen during each repetition of the experiment.

On the other hand, if one measures the direction of momentum of any single photon prior to its passage through double-slits then the momentum entangled state is collapsed. This measurement outcome reveals momentum direction of a photon on which a measurement is performed and the direction of momentum of the other photon is also revealed instantly after the collapse even without making any measurement on it. This measurement reveals which-slit path information of photons. On the other hand, which-slit path of photons in the double-double-slit can be detected by closing any single slit with a shutter. A shutter shown in Fig. 1 is considered as a photon measuring detector, if shutter is closed to block a slit $a_{2}$ and a photon is detected on screen 2 then it reveals that a photon is passed through a slit $b_{2}$ due to collapse of quantum entangled state caused by the shutter detector. As a consequence, one can find out that the other photon is passed through a slit $a_{1}$ if it is detected at $o_{1}$. Since a path of both photons is known therefore, two-photon interference is suppressed. Interesting situation appears when double-slit 2 and screen 2 are removed to allow a photon to propagate in space while other photon is passed through double-slit 1 and detected on screen 1. A single-photon interference not produced on screen 1 because of path entanglement the which-slit path information of photons can be obtained by measuring momentum of the propagating photon even after the detection of a photon on screen 1.

Furthermore, when all slits are opened and which-slit path is not detected, a photon can be detected at any location on a screen randomly during each repetition of the experiment and its detection location is not known prior to a measurement on screen. Once a photon is detected on a screen, its detection location instantly determines the amplitude to find other photon on other screen if it is not reached there. Individual photons show no interference on a screen because a well defined phase coherent amplitude to find a photon on a screen depends on a particular detection location of other photon. In this case, a single photon amplitude is completely incoherent. The information of detection location of a photon determines a particular two-photon interference pattern. In other words, in this type of joint and correlated registration of detection locations of photons, if a different detection location of a photon is selected the two-photon interference pattern exhibits a shift. If only single photons are registered on each screen without making any correlation between their detection locations then the interference pattern is not formed on each screen.

## Two-photon interference

To find out a two-photon interference in the double-double-slit experiment for a finite width of each slit, consider a source of photons located at origin is producing a two-photon quantum state $|\Psi \rangle$ as shown in Fig. 1. Double-slits can be defined by amplitude transmission functions $t_{1}(y')$ and $t_{2}(y'')$ of double-slit 1 and double-slit 2 respectively. Where $y'$ and $y''$ are the arbitrary points on double-slit 1 and double-slit 2, respectively such that the position basis states corresponding to these points located on the double-slits where a photon can be found are $|l_{1}, y'\rangle$ and $|l_{2}, y''\rangle$. Therefore, the amplitude $A_{12}$ to find photons at points $o_{1}$ and $o_{2}$ together on screens can be written as

2 $$\begin{aligned} A_{12}= \int ^{\infty }_{-\infty } \int ^{\infty }_{-\infty } \langle o_{2}| l_{2},y''\rangle t_{2}(y'') \langle l_{2},y''| \langle o_{1}|l_{1},y'\rangle t_{1}(y') \langle l_{1},y'|\Psi \rangle \mathrm {d}y' \mathrm {d}y'' \end{aligned}$$

Consider photon source has finite size and two-photon quantum state $|\Psi \rangle$ is a momentum entangled quantum state, where both the photons have same linear polarisation and frequency. Such a two-photon quantum entangled state can be produced by degenerate noncollinear SPDC with type-I phase matching in a beta-barium-borate (BBO) crystal which is pumped by a laser beam propagating along the *z*-axis (longitudinal direction), where the *z*-axis (not shown in Fig. 1) is perpendicular to the *xy*-plane (transverse plane). Photons known as the signal and the idler photons are emitted from the source with opposite momenta with nearly equal in magnitude in the transverse plane such that their two-photon momentum entangled state in the transverse momentum space is^42–46^

3 $$\begin{aligned} |\Psi \rangle = N \int \int \mathrm {d}\mathbf {q_{s}}\mathrm {d}\mathbf {q_{i}} \Phi (\mathbf {q_{s},\mathbf {q_{i}}})|\mathbf {q_{s}}\rangle |\mathbf {q_{i}}\rangle \end{aligned}$$

where $|\mathbf {q_{s}}\rangle$, $|\mathbf {q_{i}}\rangle$ are the transverse momentum quantum states of the signal and the idler photons of momentum $\mathbf {q_{s}}$ and $\mathbf {q_{i}}$, respectively and *N* is a normalisation constant. Two-photon wavefunction $\Phi (\mathbf {q_{s},\mathbf {q_{i}}})$ represents the amplitude to find a signal photon in momentum state $|\mathbf {q_{s}}\rangle$ and an idler photon in momentum state $|\mathbf {q_{i}}\rangle$. Quantum entanglement is manifested by non separability of $\Phi (\mathbf {q_{s},\mathbf {q_{i}}})$. For the pump laser beam with gaussian intensity profile of finite width in the transverse plane, the two-photon wavefunction $\Phi (\mathbf {q_{s},\mathbf {q_{i}}})$ is prominent only for momentum states of photons with opposite transverse momenta. Since source size is finite therefore, if momentum of a photon is measured precisely then the quantum state of the other photon corresponds to a momentum state of opposite momentum with finite uncertainty. Further detail on momentum entanglement of photons produced by degenerate noncollinear SPDC in the BBO crystal is given in methods.

There are two possibilities that can result in a joint detection of photons on screen 1 and screen 2. These indistinguishable possibilities are (i) the signal photon is passed through double-slit 1 and detected on screen 1 and the idler photon is passed through double-slit 2 and detected on screen 2 and (ii) the idler photon is passed through double-slit 1 and detected on screen 1 and the signal photon is passed through double-slit 2 and detected on screen 2. Any single photon (signal or idler) that can be detected after passing through the double-slit 1 is labeled as photon 1 and any single photon that can be detected after passing through the double-slit 2 is labeled as photon 2. Photon 1 and photon 2 are indistinguishable as they have same frequency and polarisation. Consider, transmission function of double-slit 1 is $t_{1}(y')= a'_{t}\left( \frac{e^{-(y'-d_{1}/2)^2/2\sigma _{1}^2}}{(2\pi )^{1/2}\sigma _{1}}+\frac{e^{-(y'+d_{1}/2)^2/2\sigma _{1}^2}}{(2\pi )^{1/2}\sigma _{1}}\right)$, which represents two gaussian slits with separation between them $d_{1}$ and slit width $\sigma _{1}$ of each slit is such that $d_{1}$ is considerably larger than $\sigma _{1}$. Similarly, transmission function of double-slit 2 is $t_{2}(y'')= a''_{t}\left( \frac{e^{-(y''-d_{2}/2)^2/2\sigma _{2}^2}}{(2\pi )^{1/2}\sigma _{2}}+\frac{e^{-(y''+d_{2}/2)^2/2\sigma _{2}^2}}{(2\pi )^{1/2}\sigma _{2}}\right)$, which represents two gaussian slits with separation between them $d_{2}$ and slit width $\sigma _{2}$ of each slit is such that $d_{2}$ is considerably larger than $\sigma _{2}$. Where, $a'_{t}$ and $a''_{t}$ are the complex multipliers of transmission functions, they include the phase shift introduced by the slits and limit the maximum transmission to one. For $a'_{t}= a''_{t}=0$, the transmission of slits is zero. Each double-slit is positioned far away from the source as compared to its slit separation. Therefore, slits are located at close inclination with the *x*-axis such that photons coming from source are incident on slits almost close to the normal incidence. To have two-photon path entanglement via the slits the double-slits are positioned such that $d_{1}/l_{1}=d_{2}/l_{2}$ and $\sigma _{1}/l_{1}=\sigma _{2}/l_{2}$. In addition, uncertainty $\Delta q_{\parallel }$ of momentum component of each photon parallel to the double-slits, provided momentum of other photon is precisely determined, is small such that $\Delta q_{\parallel }/q\ll d_{1}/l_{1}= d_{2}/l_{2}$ to suppress single photon interference by each double-slit, where *q* is the magnitude of momentum of a photon^41^. However, $\Delta q_{\parallel }/q\approx \sigma _{1}/l_{1}=\sigma _{2}/l_{2}$. These conditions implies, if a photon is passed through slit $a_{1}$ then the other photon is most likely passed through slit $b_{2}$ and if a photon is passed through slit $b_{1}$ the other photon is most likely passed through slit $a_{2}$. Therefore, photons contributing to the joint detection on screens are path entangled via the slits. However, if a photon is absorbed far away from slits at an arbitrary location $y'$ on double-slit 1 then the other photon is most probably absorbed at $y''=-y'l_{2}/l_{1}$ far away from slits of double-slit 2. Transmission of each slit is considered to be gaussian with very small width that allows a photon to pass through it. Under these considerations, the amplitude $A_{12}$ of joint detection of photons on screens gets a major contribution from a small range of momentum states of quantum state $|\Psi \rangle$. Remaining momentum states in $|\Psi \rangle$ are absorbed at double-slits. Therefore, to evaluate $A_{12}$ by using Eq. 2, a following approximation can be applied

4 $$\begin{aligned} t_{2} (y^{\prime\prime})t_{1} (y^{\prime})\langle l_{2} ,y^{\prime\prime}|\langle l_{1} ,y^{\prime}|\Psi \rangle \approx a^{\prime\prime}_{t} a^{\prime}_{t} c_{w} & \left( {e^{{iq(r_{{a1}} + r_{{b2}} )/\hbar }} \cdot \frac{{e^{{ - (y^{\prime} - d_{1} /2)^{2} /2\sigma _{1}^{2} }} }}{{(2\pi )^{{1/2}} \sigma _{1} }}\frac{{e^{{ - (y^{\prime\prime} + d_{2} /2)^{2} /2\sigma _{2}^{2} }} }}{{(2\pi )^{{1/2}} \sigma _{2} }}} \right. \\ & \quad \left. { +\, e^{{iq(r_{{b1}} + r_{{a2}} )/\hbar }} \cdot \frac{{e^{{ - (y^{\prime} + d_{1} /2)^{2} /2\sigma _{1}^{2} }} }}{{(2\pi )^{{1/2}} \sigma _{1} }}\frac{{e^{{ - (y^{\prime\prime} - d_{2} /2)^{2} /2\sigma _{2}^{2} }} }}{{(2\pi )^{{1/2}} \sigma _{2} }}} \right) \\ \end{aligned}$$

where $c_{w}$ is a constant of proportionality that depends on the two-photon wavefunction. Since photons are incident on each slit close to the normal incidence therefore, $e^{i q (r_{a1}+ r_{b2})/\hslash }$ is the two-photon amplitude of a photon to go from source to slit $a_{1}$ located at a distance $r_{a1}$ and other photon to go from source to slit $b_{2}$ located at a distance $r_{b2}$. Similarly, $e^{i q (r_{b1}+ r_{a2})/\hslash }$ is the two-photon amplitude of a photon to go from source to slit $b_{1}$ located at a distance $r_{b1}$ and other photon to go from source to slit $a_{2}$ located at a distance $r_{a2}$. The transmitted amplitude of photons via the slits $a_{1}$ and $a_{2}$ or via the slits $b_{1}$ and $b_{2}$ is negligible because $\Phi (\mathbf {q_{s},\mathbf {q_{i}}})$ is very small for these paths. Photons are path entangled via the slits and Eq. 4 represents the amplitude of transmitted photons on the double-slits that leads to the joint detection of photons.

Transmitted photon amplitude of a photon further emanates from a point on a double-slit such that it corresponds to an uniform probability distribution of the photon to be found on the screen. The amplitudes of transmitted photons to go from a point location on a double-slit to a point location on the nearest screen are $\langle o_{1}| l_{1},y'\rangle \propto e^{iq|R'|/\hslash }/|R'|^{1/2}$ and $\langle o_{2}| l_{2},y''\rangle \propto e^{iq|R''|/\hslash }/|R''|^{1/2}$ for photon 1 and photon 2, respectively. Where $R'$ and $R''$ are the distances of $o_{1}$ and $o_{2}$ from arbitrary points $y'$ and $y''$ located on double-slit 1 and double-slit 2, respectively. Since distances $s_{1}$ and $s_{2}$ of the screens from the nearest double-slits are much larger than the slit separations therefore, $\langle o_{1}| l_{1},y'\rangle \propto e^{iq(r_{1}-y'\sin (\theta _{1}))/\hslash }/r^{1/2}_{1}$ and $\langle o_{2}| l_{2},y''\rangle \propto e^{iq(r_{2}-y''\sin (\theta _{2}))/\hslash }/r^{1/2}_{2}$, where $r_{1}$ and $r_{2}$ are the distances of $o_{1}$ and $o_{2}$ from the middle points of double-slit 1 and double-slit 2, respectively as shown in Fig. 1. After solving Eq. 2 by using Eq. 4 the amplitude of joint detection of photons can be written as

5 $$\begin{aligned} A_{12}\,=\, & {} c_{n} \frac{e^{i q (r_{1}+r_{2})/\hslash } e^{i q (r_{a1}+r_{b2}+r_{a2}+r_{b1})/2 \hslash }}{(r_{1}r_{2})^{1/2}} e^{-q^{2}((\sigma _{1}\sin \theta _{1})^{2}+(\sigma _{2}\sin \theta _{2})^{2})/2\hslash ^{2}} \cos [q(d_{2}\sin \theta _{2}-d_{1}\sin \theta _{1})/2\hslash + \delta ] \end{aligned}$$

where $\delta =q (r_{a1}+r_{b2}-r_{a2}-r_{b1})/2\hslash$ and $c_{n}$ is a proportionality constant. Therefore, probability of coincidence detection $p_{12}=|A_{12}|^{2}$ of photons is

6 $$\begin{aligned} p_{12}= \frac{ |c_{n}|^{2} }{r_{1}r_{2}} e^{-q^2((\sigma _{1}\sin \theta _{1})^{2}+(\sigma _{2}\sin \theta _{2})^{2})/\hslash ^2} \cos ^{2}[q(d_{2}\sin \theta _{2}-d_{1}\sin \theta _{1})/2\hslash + \delta ] \end{aligned}$$

Probability of coincidence detection of photons is a product of two functions, where the exponential functions corresponds to a single-photon diffraction of photons from single slits and a cosine function corresponds to two-photon interference from the double-double-slit. Since photons are path entangled via the slits therefore, Eq. 6 can not be written as a product of two separate functions of variables of photon 1 and photon 2, respectively. If only the single photon detection locations on each screen are recorded without making any correlation among them then no interference pattern is formed. A single photon interference is suppressed due to quantum entanglement of paths of photons in the double-double-slit. Both photons can be detected anywhere randomly on the respective screens however, a two-photon quantum interference pattern appears only in the position correlated measurements. Probability of detection of a single photon on the respective screens can be calculated by integrating all possible paths of a single photon. However, due to quantum entanglement of paths this integral results in an addition of probability of detection of a single photon via each slit of a double-slit. Therefore, probabilities $p_{1}$ and $p_{2}$ to find a single photon on screen 1 and screen 2 are

7a $$\begin{aligned} p_{1}\propto \frac{1}{r_{1}} e^{-q^2(\sigma _{1}\sin \theta _{1})^{2}/\hslash ^2} \end{aligned}$$

7b $$\begin{aligned} p_{2}\propto \frac{1}{r_{2}} e^{-q^2(\sigma _{2}\sin \theta _{2})^{2}/\hslash ^2} \end{aligned}$$

where each probability distribution of a single photon detection is gaussian and single photon interference pattern is not exhibited.

Actual experiment is performed in the three-dimensional position space, where momentum of photons and distances of detectors from double-slits are measured in the three-dimensional position space. Therefore, projection of momentum and distances onto the transverse plane should be considered in order to be consistent with Eqs. 6 and 7. In actual experiment the slits are located parallel to the transverse plane, detector displacement is parallel to the transverse plane and displacement range is such that $y_{1}\ll s_{1}$, $y_{2}\ll s_{2}$ therefore, $\sin {\theta _{1}}\sim y_{1}/s_{1}$ and $\sin \theta _{2}\sim y_{2}/s_{2}$. Under these considerations, terms in the form of a ratio, of transverse momentum and distance of a screen from a corresponding double slit, appears in Eqs. 6 and 7. Therefore, photon momentum and distances of detectors from double-slits measured in the three-dimensional position space can be placed in these equations to calculate the patterns.

## Experiment

**Figure 2:**
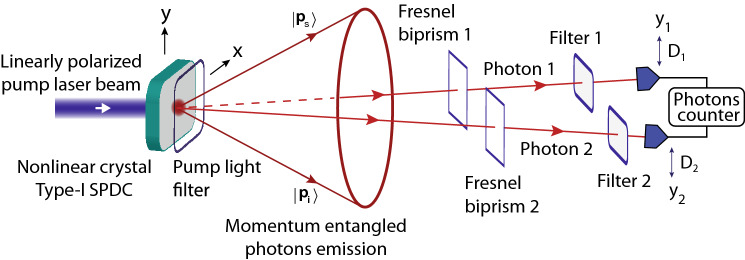
A schematic diagram of the experimental configuration of the double-double-slit experiment. Momentum entangled photon pairs are produced in a conical emission pattern by a nonlinear crystal. A double-double-slit configuration is realised with two Fresnel biprisms.

Double-double-slit experiment presented in this paper is performed with momentum entangled photons produced by type-I degenerate noncollinear SPDC^21,24,39,42–45,47^. A BBO crystal is pumped by an extraordinary linearly polarised laser beam of wavelength 405 nm and down converted photon pairs of wavelength 810 nm with ordinary polarisation are produced in the forward direction in a conical emission pattern according to momentum and energy conservation as shown in Fig. 2. To produce a virtual double-double-slit configuration, two Fresnel biprisms are placed in the path of photons and photons are detected by single photon avalanche photodetectors $D_{1}$ and $D_{2}$. Optical narrow band pass filters are placed in front of each photon detector to stop the background light. Down converted photons have same frequency and linear polarisation, which is perpendicular to the polarisation of the pump laser beam. Pump laser intensity is such that probability of more than single photon pair production is extremely small. Number of photon counts of each single photon detector and their mutual coincidence photon counts are measured with a two channel single photon counting module. Transverse mode extension of the pump laser beam is reduced to keep the source size much smaller than the slit separation but it is large so that momentum entanglement of photons is preserved.

**Figure 3:**
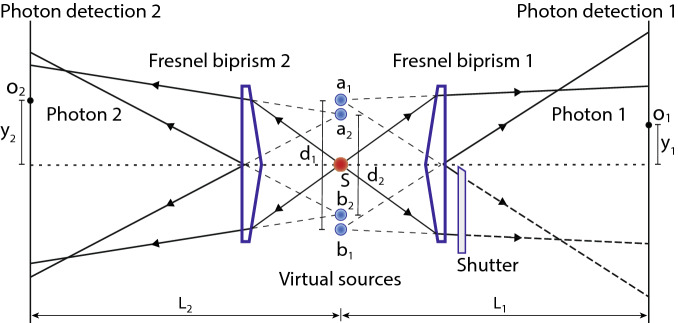
An unfolded diagram of the double-double-slit experiment realised with Fresnel biprisms. Virtual sources correspond to virtual slits. To detect which-slit path information of photons, a shutter can be placed in a path of photon 1 in such a way that a virtual slit $b_{1}$ is blocked.

**Figure 4:**
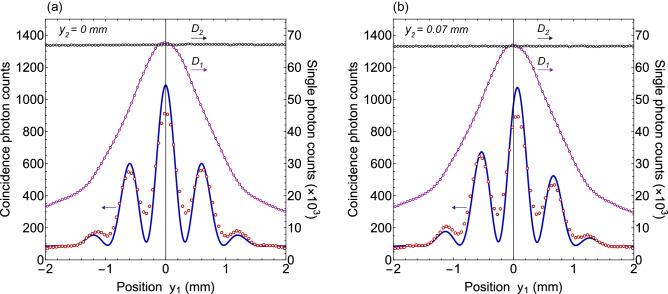
Two-photon interference pattern obtained by measuring the coincidence photon counts when measurement location $y_{2}$ of photon 2 is stationary. Experimental measurements are represented by open circles and solid line interference pattern is the two-photon interference calculated from theory. There is no interference exhibited by the individual photons as shown by single photon counts of single photon detectors $D_{1}$ and $D_{2}$. Where (**a**) for $y_{2}=~0~{\text {mm}}$ and (**b**) for $y_{2}=~0.07~{\text {mm}}$. Two-photon interference pattern is shifted as the location $y_{2}$ of photon detector $D_{2}$ is displaced.

**Fig. 5:**
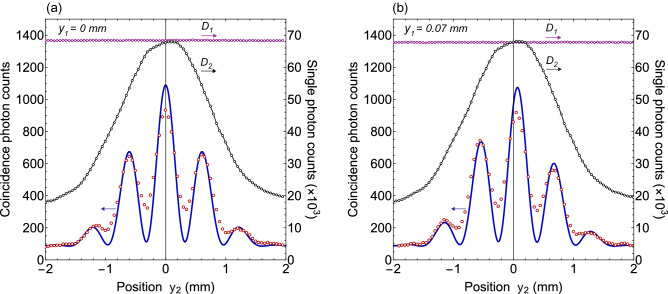
Two-photon interference pattern obtained by measuring the coincidence photon counts when measurement location $y_{1}$ of photon 1 is stationary. Experimental measurements are represented by open circles and solid line interference pattern is the two-photon interference calculated from theory. There is no interference exhibited by the individual photons as shown by single photon counts of single photon detectors $D_{1}$ and $D_{2}$. Where (**a**) for $y_{1}=~0~{\text {mm}}$ and (**b**) for $y_{1}=~0.07~{\text {mm}}$. Two-photon interference pattern is shifted as the location $y_{1}$ of photon detector $D_{1}$ is displaced.

For type-I phase matching, the BBO emits two degenerate photons with opposite transverse momenta in the transverse plane as shown in Fig. 2 and quantum state of photons of a pair corresponds to a continuous variable momentum entangled quantum state. A two-dimensional unfolded diagram of the experimental schematic given in Fig. 2 is shown in Fig. 3, where a source *S* positioned at origin is a BBO crystal that emits momentum entangled photons pairs. Two virtual double-slits are realised with two Fresnel biprisms positioned in the path of both photons. Fresnel biprisms are aligned in such a way that after passing through each Fresnel biprism, paths of a photon can be extrapolated in the backward direction such that it appears as if the photon is originated from two virtual sources which are considered as slits. Each Fresnel biprism produces a virtual double-slit with gaussian slits of finite size. In this way, a double-double-slit configuration is realised with slit separation $d_{1}$ and $d_{2}$ of a virtual double-slit 1 and a virtual double-slit-2, respectively as shown in Fig. 3. The virtual double-double-slit is parallel to the transverse plane and both photon detectors are displaced parallel to the transverse plane. Photon 1 is detected at location $o_{1}$ and photon 2 is detected at location $o_{2}$ by single photon detectors. Shortest distance of $D_{1}$, $D_{2}$ are $L_{1}$, $L_{2}$ from double-slit 1 and double-slit 2, respectively as shown in Fig. 3. Single photon counts of single photon detectors positioned at different locations $y_{1}$ and $y_{2}$ and the corresponding coincidence photons counts are recorded. Experimental results on the double-double-slit interference of momentum entangled photons are shown in Fig. 4, where the coincidence and single photon counts of photons are measured at different $y_{1}$ positions of single photon detector $D_{1}$ when single photon detector $D_{2}$ a kept stationary at a location $y_{2}$. Single photon counts of each single photon detector and the coincidence photon counts are presented by open circles in Fig. 4a for $y_{2}$= 0 mm where each data point is the mean of photon counts acquired for 5 s and twenty five repetitions of the experiment. The coincidence photon counts represent a two-photon interference pattern and the corresponding theoretically calculated interference given by Eq. 6 with a consideration of finite size of photon detectors is shown by a solid line. Effect of finite size of detectors raises the minima of the interference pattern. Single photon counts show no interference pattern as presented by the theoretical analysis also. According to the experimental considerations, $\sin \theta _{1}\sim y_{1}/s_{1}$ and $\sin \theta _{2}\sim y_{2}/s_{2}$. The coincidence interference pattern exhibits a shift when measurement location $y_{2}$ of photon 2 is shifted to another position by displacing single photon detector $D_{2}$. A shift in the two-photon interference pattern is shown in Fig. 4b for $y_{2}$= 0.07 mm. In the opposite case, photon 1 is detected at a stationary location $y_{1}$ and photon 2 is detected at different locations $y_{2}$. Results of the coincidence measurements of photon counts and single photon counts are shown in Fig. 5a for $y_{1}$= 0 mm and Fig. 5b for $y_{1}$= 0.07 mm. Solid line in each plot of coincidence measurements is the two-photon interference calculated from Eq. 6 by including the effect of finite size of detectors. A two-photon interference shows a shift with the displacement of position of detection location $y_{1}$ of photon 1, while single photon counts show no interference as theoretically shown in the previous section. In the experiment, each virtual double-slit has a same slit separation $d_{1}=d_{2}=~0.67~\hbox {mm}$ and $L_{1}=L_{2}=528$ mm.

**Fig. 6:**
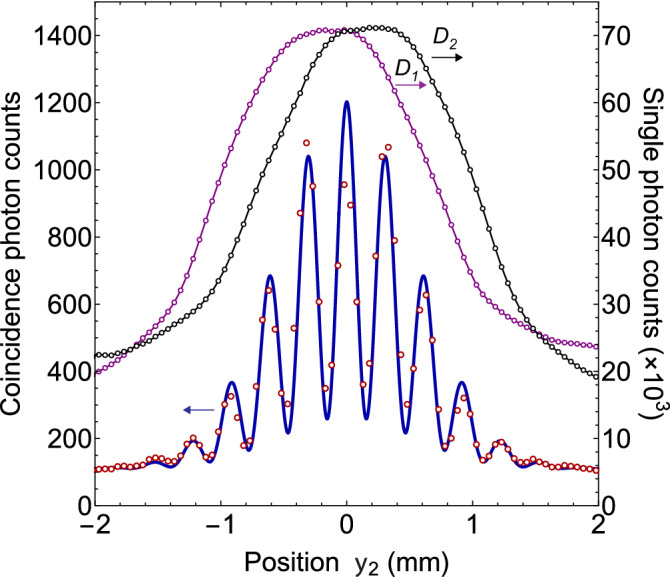
In this experiment both the single photon detectors $D_{1}$ and $D_{2}$ are displaced in the opposite direction such that $y_{2}=-y_{1}$. Fringe separation of two-photon interference is reduced and individual photons exhibit no interference.

It is evident from the probability of coincidence photon detection given in Eq. 6 that for $d_{1}=d_{2}$, the fringe separation of the two-photon interference pattern will reduce to half if the coincidence photon counts are measured for $y_{2}=-y_{1}$ i.e. when both single photon detectors are displaced in the opposite direction. For this case, a two-photon interference pattern and a single photon pattern are shown in Fig. 6, where each measured data point of photon counts is the mean of data acquired for 5 s and twenty five repetitions of the experiment. It is a different experimental set-up than the previous case and in this case $d_{1}=d_{2}=~0.682~\hbox {mm}$ and $L_{1}=L_{2}=520$ mm. Solid line represents a theoretically calculated two-photon interference by including the effect of finite size of photon detectors. It is evident that the fringe separation is reduced to half and therefore, the number of fringes are increased within the same gaussian envelop. There is no formation of coincidence interference pattern if both the single photon detectors are displaced in the same direction such that $y_{2}=y_{1}$ as it is evident from Eq. 6.

### Detection of which-slit path of photons

In the double-double-slit experiment, photons are momentum entangled and they can reveal the which-slit path information of each other if one of them is detected close to any double-slit. If one blocks a single slit of a double-slit then the which-slit path can be detected from the coincidence detection of photons. Consider a slit $a_{2}$ is blocked by closing a shutter shown in Fig. 1.

**Fig. 7:**
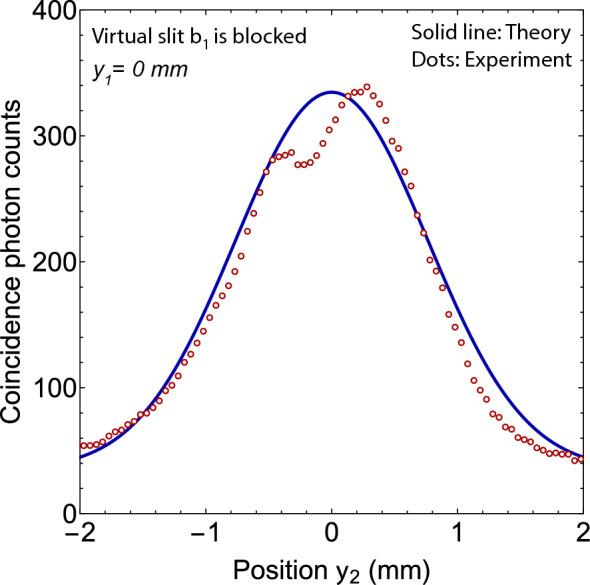
Two-photon coincidence pattern when a virtual slit $b_{1}$ is blocked. It is evident that two-photon interference is suppressed. Solid line is a theoretically calculated two-photon pattern.

If photons of a single pair are detected on screen 1 and screen 2 together then it is evident that photon 2 has passed through slit $b_{2}$. One can consider a path blocking shutter as another single photon detector $D_{3}$. If $D_{3}$ detects a photon 2 then the path entangled state of photons collapses and the which-slit path of photon 1 in the double-slit 1 is also determined. In this case, the which-slit path of photon 1 is through the single slit $b_{1}$. Since each photon is passed through a single slit therefore, neither a single photon nor a two-photon interference of joint detections of photons on screen 1 and a path blocking single photon detector $D_{3}$ will occur. On the other hand, if photon 2 is detected on screen 2 then the path entangled state is collapsed by $D_{3}$ such that photon 2 is passed though slit $b_{2}$ and photon 1 is passed through slit $a_{1}$. Single photon detection probability of photon 2 on screen 2 will reduce by half in comparison to the case when both slits were open. Probability of a single photon detection of photon 1 on screen 1 will remain unchanged because detection of photon 1 does not reveal any information whether a photon 2 is detected at screen 2 or by $D_{3}$. In the experiment, shutter is placed after the Fresnel biprism 1 such that a virtual slit $b_{1}$ shown in Fig. 3 is blocked. This configuration resembles to a double-double-slit schematic shown in Fig. 1 where the slit $a_{2}$ can be blocked by a shutter. Photon counts are measured for different locations $y_{2}$ of single photon detector $D_{2}$ by keeping single photon detector $D_{1}$ stationary at $y_{1}$. Experimental results of a path detection experiment are shown in Fig. 7. Experimental parameters in this case are same as for the experiment described in the previous section. It is evident from the experimental results, if a which-slit path information of photons is extracted by blocking any single slit then both single and two-photon interferences are suppressed.

## Discussion

This paper has presented experimental and conceptual insights on the quantum double-double-slit thought experiment first introduced by Greenberger, Horne and Zeilinger^17^. Experiments presented in this paper are performed with momentum entangled photons produced by type-I degenerate noncollinear SPDC process in a BBO crystal. In the experiment, once both photons traverse the respective double-slits, they can be detected anywhere on screens randomly because when a photon strikes a screen its quantum state collapses to one location randomly. Patterns emerge in many repetitions of the same experiment. Since paths of photons in the double-double-slit configuration are quantum entangled, their individual quantum states are phase incoherent therefore, formation of a single photon interference is suppressed. However, if a photon is detected on a screen at a well defined location, the quantum state of other photon, which is not detected, corresponds to a phase coherent amplitude to find it on second screen. Therefore, knowledge of detection locations of a photon labels the different phase coherent amplitudes to find other photon on second screen. However, in subsequent repetitions of the experiment, detection locations of photons can vary randomly. For a given location of detection of a photon the other photon shows interference pattern which corresponds to the conditional interference pattern of two photons. As a detection location of a photon is varied the conditional interference pattern is shifted. On the other hand, if no correlations of detection locations of photons are made then there is no way to select a particular phase coherent amplitude in repeated measurements. Eventually, a single photon interference pattern does not appear. It is also shown experimentally and conceptually, if a which-slit path information of any one of the photons is detected then a single photon interference and a two-photon interference disappear because of random collapse of quantum superposition of paths.

## Methods

### Two-photon momentum entangled state

Two-photon momentum entangled state is produced by a negative uniaxial second order nonlinear BBO crystal by type-I SPDC process. A pump photon of frequency $\omega _{p}$ is split into two photons known as the signal photon and the idler photon of frequency $\omega _{s}$ and $\omega _{i}$, respectively. A linearly polarised extraordinary pump laser beam propagating along the *z*-axis is incident on the crystal. A planar surface of the crystal is in the *xy*-plane with origin at the centre, where $l_{x}$, $l_{y}$, $l_{z}$ are the spatial extensions of the crystal along each axis. Ordinary photons produced by SPDC are linearly polarised with propagation vectors in three dimensions $\mathbf {k_{s}}$ and $\mathbf {k_{i}}$. In type-I phase matching, due to dispersion and anisotropy of the crystal, the signal and the idler photons are produced with non zero angle of their propagation vectors with the propagation vector $\mathbf {k_{p}}$ of the pump laser beam to conserve momentum of photons. For a thin crystal and a narrow pump laser beam, it produces a conical emission pattern of down converted photons. Pump laser beam is considered to be a continuous beam and due to low down conversion efficiency the pump laser beam amplitude is considered to be constant. The amplitude to produce more than one photon pair is extremely small, which is desirable in experiments with a single quantum entangled pair of photons during each cycle of the experiment. Pump laser beam is considered to be monochromatic, frequencies of the signal and the idler photons are same in the experiment and their propagation vectors are making a nonzero angle with the propagation direction of pump photons. Narrow band pass filters are placed after the crystal and prior to the detectors to increase coherence length. Due to sufficiently long interaction time, energy conservation condition is fulfilled such that $\hslash \omega _{p}=\hslash \omega _{s}+\hslash \omega _{i}$. Since polarisation of down converted photons is same therefore, two-photon quantum state produced by degenerate type-I noncollinear SPDC process can be written as^42–47^

8 $$\begin{aligned} |\Psi \rangle _{spdc}\approx c_{0}|0\rangle +c_{1} \int \int \mathrm {d}\mathbf {p_{s}}\mathrm {d}\mathbf {p_{i}} \Phi (\mathbf {p_{s},\mathbf {p_{i}}})|\mathbf {p_{s}}\rangle |\mathbf {p_{i}}\rangle \end{aligned}$$

where $c_{0}$, $c_{1}$ are complex coefficients, $c_{1}$ depends on the pump laser beam intensity and second order nonlinear coefficient of the crystal. The quantum states $|\mathbf {p_{s}}\rangle$ and $|\mathbf {p_{i}}\rangle$ represent single photon momentum states of the signal and the idler modes of momentum vectors $\mathbf {p_{s}}=\hslash \mathbf {k_{s}}$ and $\mathbf {p_{i}}=\hslash \mathbf {k_{i}}$, respectively. The quantum state $|0\rangle$ is a vacuum state of the signal and the idler modes without any photon. A two-photon wavefunction $\Phi (\mathbf {p_{s},\mathbf {p_{i}}})$ in the momentum space can be written as

9 $$\begin{aligned} \Phi (\mathbf {p_{s},\mathbf {p_{i}}})=c_{p}\int \mathrm {d}\mathbf {q_{p}} \mathbf {\nu }(\mathbf {q_{p}}) {{\,\mathrm{sinc}\,}}\left( \frac{\Delta p_{x}l_{x}}{2\hslash }\right) {{\,\mathrm{sinc}\,}}\left( \frac{\Delta p_{y}l_{y}}{2\hslash }\right) {{\,\mathrm{sinc}\,}}\left( \frac{\Delta p_{z}l_{z}}{2\hslash }\right) \end{aligned}$$

where $c_{p}$ is a constant and the integration is carried out in transverse momentum space which is a projection of three-dimensional momenta onto the transverse two-dimensional *xy*-plane, $\Delta p_{j}$ = $p_{sj}+p_{ij}-p_{pj}$ for $j\in \{x,y,z\}$ and $p_{sj}$, $p_{ij}$, $p_{pj}$ represent components of momentum of the signal, the idler and pump photons along the *j*-axis, respectively. A function $\mathbf {\nu }(\mathbf {q_{p}})$ is the normalised amplitude of pump laser beam corresponding to momentum projection $\mathbf {q_{p}}$ in the transverse plane. For a plane wave, $\mathbf {\nu }(\mathbf {q_{p}})$ is a Dirac delta function. If crystal extensions $l_{x}$ and $l_{y}$ are much larger than the wavelength of pump laser beam then $\Delta p_{x}$ and $\Delta p_{y}$ should be very small otherwise $\Phi (\mathbf {p_{s},\mathbf {p_{i}}})$ diminishes. Therefore, $\mathbf {q_{p}}$=$\mathbf {q_{s}}+\mathbf {q_{i}}$ for transverse momentum $\mathbf {q_{s}}$ of the signal photon and transverse momentum $\mathbf {q_{i}}$ of the idler photon. It corresponds to conservation of transverse momentum of photons. For a gaussian transverse momentum profile of the pump laser beam with radius $\sigma _{p}$ in the position-space and for a very small angle between the pump photon momentum and the signal photon or the idler photon momentum, the two-photon wavefunction is given in Ref.^44^,

10 $$\begin{aligned} \Phi (\mathbf {q_{s},\mathbf {q_{i}}})= c_{\Phi }{{\,\mathrm{sinc}\,}}\left( \frac{l_{z}}{4 \hslash ^{2}|\mathbf {k_{p}}|} |\mathbf {q_{s}}-\mathbf {q_{i}}|^{2}\right) {{\,\mathrm{e}\,}}^{-\sigma ^{2}_{p}|\mathbf {q_{s}}+\mathbf {q_{i}}|^{2}/\hslash ^{2}} \end{aligned}$$

where, $c_{\Phi }$ is a constant of proportionality. Two-photon wavefunction $\Phi (\mathbf {q_{s},\mathbf {q_{i}}})$ is prominent if transverse momenta of photons are equal and opposite to each other.

Two-photon quantum entangled state in the transverse momentum space can be written as

11 $$\begin{aligned} |\Psi \rangle = N \int \int \mathrm {d}\mathbf {q_{s}}\mathrm {d}\mathbf {q_{i}} \Phi (\mathbf {q_{s},\mathbf {q_{i}}})|\mathbf {q_{s}}\rangle |\mathbf {q_{i}}\rangle \end{aligned}$$

where *N* is a normalisation constant and the vacuum state is not relevant in the context of present experiment. In general the momentum entanglement is manifested by non separability of two-photon wavefunction $\Phi (\mathbf {q_{s},\mathbf {q_{i}}})$.

### Experimental details

A linearly polarised pump laser light of wavelength 405 nm of gaussian beam profile is incident on a BBO crystal at room temperature. Two orthogonally polarised momentum entangled photons of wavelength 810 nm are emitted in a conical emission pattern and single photons are detected by avalanche single photon detectors. Each photon pair is passed through two Fresnel biprisms to realise a virtual double-double-slit configuration. After the crystal, pump light at 405 nm is blocked by an optical band pass filter with transmission window peak at 810 nm where the full-width-half-maximum of the transmission window is about 10 nm. Two multimode optical fibers carry photons from points $o_{1}$ and $o_{2}$ to each single photon detector. The other end of each optical fiber is mounted on separate three-dimensional precision displacement stages and photons are coupled to each optical fiber with an objective lens. Narrow apertures are positioned at $o_{1}$ and $o_{2}$ prior to the objective lens to allow photons to be detected at these two points only. Prior to each fiber coupler two optical band pass filters (filter 1 and filter 2) are placed in the path of each photon to block scattered photon of wavelength 405 nm and background photons reaching each single photon detector. Photon correlations are measured by counting electrical pulses produced by each single photon detector. Experimentally measured coincidence and single photon counts are shown by open circles data points in the figures. Each data point is acquired for 5 s with twenty five repetitions of the same experiment. Experimental results are compared with theoretical calculations considering the effect of finite size of photon detectors.
